# Comparison of Antioxidant and Anti-Inflammatory Effects of Honey and Spirulina platensis with Sulfasalazine and Mesalazine on Acetic Acid-Induced Ulcerative Colitis in Rats

**DOI:** 10.31661/gmj.v8i0.1095

**Published:** 2019-06-10

**Authors:** Nadia Rezaei, Mohammad Hassan Eftekhari, Nader Tanideh, Maral Mokhtari, Zahra Bagheri

**Affiliations:** ^1^Department of Clinical Nutrition, School of Nutrition and Food Sciences, Shiraz University of Medical Sciences, Shiraz, Iran; ^2^Department of Pharmacology, School of Medicine, Shiraz University of Medical Sciences, Shiraz, Iran; ^3^Department of Pathology, School of Medicine, Shiraz University of Medical Sciences, Shiraz, Iran; ^4^Department of Biostatistics, School of Medicine, Shiraz University of Medical Sciences, Shiraz, Iran

**Keywords:** Spirulina, Honey, Ulcerative Colitis, Antioxidant, Oxidative Stress

## Abstract

**Background:**

Antioxidant therapy has gained attention for the treatment of ulcerative colitis (UC). The excessive generation of reactive oxygen/nitrogen species in the gastrointestinal tract increases oxidative stress, thereby leading to antioxidant defense depletion, lipid peroxidation, inflammation, tissue damage, and ulceration. Spirulina platensis (SP) and honey are excellent sources of potent antioxidants such as polyphenols and other bioactive compounds. We aimed to investigate antioxidant and anti-inflammatory effects of honey and SP in comparison with sulfasalazine (SSZ) and mesalazine on acetic acid-induced colitis (AA-colitis) in rats.

**Materials and Methods:**

Fifty-six Sprague Dawley male rats were allocated to seven groups, with each group comprising eight rats. UC was induced, except in normal controls (NC). All groups received oral treatments for seven days. The normal saline solution of 2 mL was intrarectally administered to the NC group. The AA-colitis and NC groups received 2 mL acetic acid intrarectally as a single dose and 2 mL normal saline for seven consecutive days orally. The mesalazine group received 100 mg/kg mesalazine, the SSZ group 360 mg/kg SSZ, the honey or H group 1 mL honey diluted with 1 mL distilled water, the SH group 1g/kg SP and 1 mL honey, and the SP group 1g/kg SP. After clinical activity score assessment, the rats were sacrificed. Colonic weight/length ratio, prostaglandin E2 (PGE2), myeloperoxidase (MPO), nitric oxide (NO), malondialdehyde (MDA), interleukin-1β (IL-1β), tumor necrosis factor-α (TNF-α), interleukin-6 (IL-6), glutathione peroxidase (GPx), total antioxidant capacity (TAC), reduced glutathione (GSH), and superoxide dismutase (SOD) were measured. Colonic histopathological changes were observed microscopically.

**Results:**

Treatment of UC with SP, honey, and combination regimen significantly reduced TNF-α, IL-1β, IL-6, MDA, MPO, NO, and PGE2, and increased TAC, GSH, GPx, and SOD in interventional groups compared to the AA-colitis group (P<0.05).

**Conclusion:**

Honey and SP might be beneficial food supplements for medical nutrition therapy in UC.

## Introduction

Ulcerative colitis (UC), a major form of chronic inflammatory bowel disease (IBD), is described as relapsing inflammation and ulceration of colon and rectum [1, 2]. Its exact etiology is not completely understood, but there is an agreement that abnormal immune response, environmental factors, and genetics are responsible for UC onset and progression [3]. In the last decades, UC prevalence and incidence have increased in Asian countries on account of extensive changes in people’s lifestyle, particularly their dietary habits [4]. Traditional Asian diets are rich in flavonoids that have protective effects against colon diseases [5].UC severely decreases the patient’s quality of life and may result in colorectal cancer [6], with toxic megacolon and severe bleeding being its acute complications and epithelial dysplasia or cancer its chronic complications [7]. Oxidative stress plays an important role in the initiation and progression of pathological conditions such as IBD, gastrointestinal ulcers, and cancers. Normal metabolism and cellular respiration produce reactive oxygen species (ROS) in a balanced manner, which is not harmful. In the gastrointestinal tract, endogenous and exogenous agents such as alcohol, certain foods, nonsteroidal anti-inflammatory drugs, cigarette smoking, chemical factors, and inflammatory status (such as injury, ischemia, and infections) stimulate the overproduction of ROS, thereby disrupting the oxidant-antioxidant balance of the mucosa. Also, harmful substances stimulate inflammatory cells, including macrophages and neutrophils, resulting in the production of proinflammatory cytokines and other mediators that cause oxidative stress [8]. Pro-inflammatory cytokines and ROS lead to membrane lipid peroxidation, which causes reduction of epithelial barrier integrity and intestinal damage in IBD [9].Intrarectal administration of acetic acid in the animal model of colitis disrupts the balance of mucosal antioxidant defense. Acid releases protons that result in massive epithelial damage. Infiltration of inflammatory cells, especially neutrophils, leads to the generation of superoxide anion, hydroxyl radicals, and several reactive species. These processes are accompanied by substantial necrosis of intestinal layers, vascular dilation, submucosal ulceration, and edema that have a remarkable similarity to human colitis [10]. Previous studies have shown that the administration of acetic acid (AA) in experimental colitis causes a significant increase in malondialdehyde, a lipid peroxidation end product. Inflammatory factors, including myeloperoxidase, prostaglandin E2, nitric oxide, and proinflammatory cytokines reduce antioxidant markers such as superoxide dismutase, glutathione peroxidase, and glutathione [2, 6].Therapeutic approaches aim to induce and maintain remission and to improve the patient’s nutritional status and quality of life [11]. At present, the principal drugs used for treating UC depend on disease severity, its complications, and the clinical goal; the drug classes include anti-inflammatory and immunomodulatory drugs (methotrexate, azathioprine, mercaptopurine), corticosteroids (prednisolone), 5-aminosalicylic acid, anti-tumor necrosis factor-α (TNF-α) antibodies, and sometimes antibiotics [12, 13]. Although these drugs are effective and have shown good therapeutic outcomes, their adverse effects such as elevated blood pressure, abdominal pain, cramps, and diarrhea have limited their application [14]. In recent years, dissatisfaction with these medications has led researchers to look for and evaluate natural products that possess antioxidant and anti-inflammatory characteristics as complementary and alternative therapies. Natural dietary products such as honey and Spirulina platensis safely suppress inflammation with no side effects, in addition to being more effective and inexpensive [15, 16].Honey possesses strong antioxidant, anti-inflammatory, and antimicrobial properties, which is due to the presence of phenolic acids, flavonoids, enzymes, vitamins, minerals, and other bioactive components. Furthermore, it has wound-healing properties and regenerative effect on damaged tissues through the movement of stem cells [17]. Several studies have reported the effectiveness of honey in improving inflammatory disorders, for example, the experimental model of colitis, atherosclerosis [17], gastric ulcers [15], bedsores, and skin ulcers [18]. Over the last few decades, SP, an edible photosynthetic microalga belonging to phylum cyanobacteria, has received much attention due to its novel functional phytonutrients and pharmaceutical compounds [19]. Spirulina is an excellent source of nutrients such as essential fatty acids, high biological value proteins, minerals, and vitamins. It also possesses strong antioxidants, including C-phycocyanin, vitamins E and C, β-carotene, phenolic compounds, γ-linolenic acid, ergothioneine, zeaxanthin, selenium, zinc, and α-lipoic acid [2, 20-23]. Several in vitro and in vivo investigations exhibited antioxidant, anti-inflammatory, antiallergic, immunomodulatory, antimicrobial (i.e., antibacterial, antiviral, and antifungal), and anticancer properties of spirulina [24], including its therapeutic effects on several inflammatory disorders, for example, arthritis, colitis, and cardiovascular diseases [2, 25]. So far, only one study has reported the effect of oral spirulina on experimental colitis [26]. Few studies were conducted to examine the therapeutic effect of honey on colitis when administered orally [17, 27, 28]. One study assessed the effect of honey on the colonic tissue. A macroscopic view revealed changes in the tissue [29]. All investigations measured a small number of inflammatory parameters and antioxidant biomarkers.This study aimed to investigate the antioxidant and anti-inflammatory effects of honey and SP in comparison with those of sulfasalazine (SSZ) and mesalazine on UC induced by AA in rats. For the first time, we examined the impact of co-administration of two natural foods—honey and SP—on oxidative stress and immunomodulatory markers, as well as antioxidant status and histopathological changes in comparison with SSZ and mesalazine in experimental colitis. Hence, we aimed to investigate the antioxidant and anti-inflammatory effects of honey and SP in comparison with SSZ and mesalazine on UC induced by acetic acid in rats.

## Materials and Methods

### Materials and Reagents

Pure Hawaiian SP was obtained from Cyanotech Corporation, Kailua-Kona, HI, USA. Honey was purchased from Khorramabad, Lorestan Province, Iran. Mesalazine and SSZ were purchased from Daroupakhsh Distribution Co., Tehran, Iran. Rat PGE2, MPO, Elisa kits, GPx, GSH, MDA, SOD, TAC, and NO assay kits were bought from ZELBio GmbH, Germany; rat interleukin-6 (IL-6), IL-1β, and TNF-α ELIZA kits were obtained from Diacolone SAS, France. In this study, analytical grade chemicals were used. All laboratory devices were calibrated before usage.

### Experimental Design

Fifty-six male Sprague Dawley rats were bought from the animal house from Shiraz University of Medical Sciences, Shiraz, Iran. For adaptability, rats (weighing 200-240 g) were housed in a room at constant environmental conditions (22±2°C, 12-hour light/dark cycle, and 55±10 percentage humidity) 1 week before the commencement of the intervention. Animals had free access to water and food. Next, they were divided into seven groups by a person who was blind to the procedure (n=8). All groups received their respective treatments for seven consecutive days by oral gavage, once daily in the morning, from the first day, 2 hours after colitis induction. The groups were as follows:

1. Normal control (NC) group: Rats in this group received 2 mL normal saline administered intrarectally as a single dose and 2 mL normal saline administered orally for seven consecutive days.
2. AA-colitis group: Rats in this group received 2 mL AA (3% v/v) by enema as a single dose and 2mL normal saline orally for seven consecutive days.
3. Colitis + mesalazine group: Rats in this group received 100 mg/kg mesalazine.
4. Colitis + SSZ group: Rats in this group received 360 mg/kg SSZ.
5. Colitis + honey (H) group: Rats in this group received 1 mL honey diluted with 1 mL distilled water.
6. Colitis + SP + honey or SH group: Rats in this group received 1 mL honey diluted with 1 mL distilled water in the morning and 1g/kg SP (2 mL suspension with distilled water) orally in the afternoon.
7. Colitis + SP group: Rats in this group received 1g/kg SP (2 mL suspension with distilled water).

Scores of bleeding, stool consistency, and weight loss were summed up, then the total was divided into three, and the total clinical scores (TCS) were calculated at 48 hours after the colitis induction per Thippeswamy *et al*. criteria [30]. On the eighth day, rats were anesthetized using ethyl ether, and serum specimens were collected and stored at –75ºC for biochemical estimations. Thereafter, the rats were sacrificed. Their abdomen was opened, colon cut longitudinally, and then slowly washed with cold saline. Thereafter, the colonic adherent adipose tissue was eliminated, and the colon was dried on filter paper. The weight and length of the entire colon were measured for calculating the weight/length ratio.

### Colitis Induction

After the acclimatization period, for 36 hours, rats were not given food; however, they could freely drink water. Then they were anesthetized with ethyl ether inhalation. A soft polyethylene tube of 2-mm diameter was used to inject 2 mL (3%, v/v) acetic acid into the 8-cm depth of the distal colon through the rectum. To prevent acid leakage, the rats were hanged from the tail for 30 seconds.

### Histological Evaluation

Fixed colonic tissue (2 cm) in 10% buffered formalin was used for histological evaluation. Embedded specimens were cut into sections of 5-µm thickness in paraffin wax blocks, and then hematoxylin and eosin (H&E) staining were performed. A pathologist, blind to study, observed histopathological changes microscopically and scored them following the Dieleman *et al*. criteria [31]. The remaining portions of colonic specimens were stored at –75 ºC for further biochemical examinations.

### Biochemical Analysis

Colonic tissue was homogenized; MPO, MDA, NO, and PGE2 concentrations in homogenized supernatants were assessed using ELISA kits as instructed by the manufacturer. The GPx and SOD activity, TNF-α, IL-1β, and IL-6 concentrations, serum GSH, and TAC levels were evaluated according to the manufacturer’s instructions.

### Ethics Statement

The local ethics committee of Shiraz University of Medical Sciences approved this study (number: IR.SUMS.REC.1394.S605). All efforts were made to minimize animal pain or suffering during experimentation according to relevant guidelines published about the use of laboratory animals.

### Statistical Analysis

Data were presented as mean ± standard error of measurement. The analysis was performed using the GraphPad Prism version 6.07 (GraphPad software, San Diego, CA) and SPSS version 16 statistical software (SPSS Inc.,Chicago, USA). The nonparametric Kruskal–Wallis test with the Dunn test as post hoc comparison was used to analyze data that did not have a normal distribution. P<0.05 was accepted as significant.

## Results

**Figure 1:**
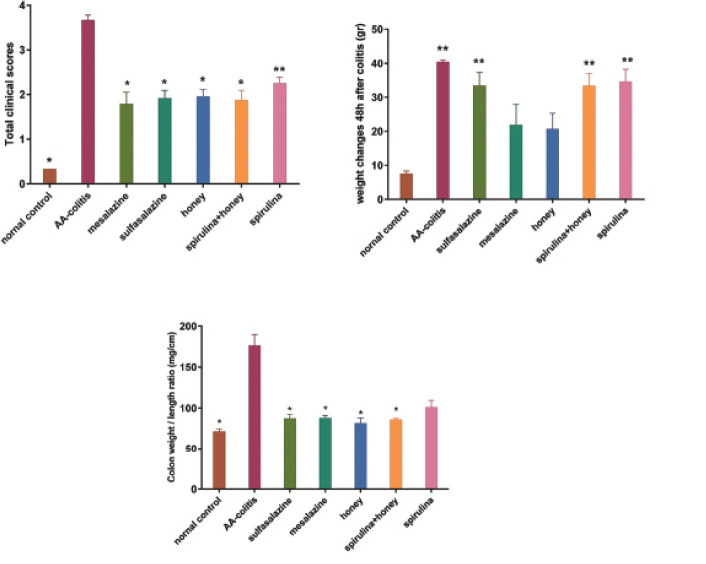
Effects of honey, Spirulina platensis, and S. platensis + honey on colon weight/length ratio, TCS, and weight loss. Results are expressed as mean ± SEM. *P<0.05 vs. AA-colitis group, **P<0.05 vs. normal control group

Colitis severity was measured based on TCS after 48 hours of colitis induction. Honey alone and co-administration of honey and SP significantly reduced TCS when compared with AA-colitis group (P=0.037 and P=0.038, respectively). Colonic weight/length ratio of all treated groups, except SP, significantly decreased in comparison with the AA-colitis group (Figure-1). Colitis induction increased serum IL-6, TNF-α, and IL-1β by 5-, 4.25-, and 1.9-fold, respectively in AA-colitis group in comparison to the NC group. Treatment with SSZ, mesalazine, honey, SP and combination regimen reversed alterations in these biochemical biomarkers. The MPO levels of colonic tissues are shown in Table-1. All treatments significantly decreased the MPO level in colonic tissues in comparison with AA-colitis group. Honey and combination regimen, similar to mesalazine and SSZ, attenuated colonic tissues,NO production in comparison with AA-colitis group. The NO level increased by fourfold in the AA-colitis group when compared to the NC group.Colonic tissue MDA level in the colitis groups that received honey, SP, and combination regimen similar to mesalazine- and SSZ-treated groups significantly improved in comparison with the AA-colitis group. Colonic tissues’ PGE2 level decreased in all treated colitis groups compared to AA-colitis group. After the induction of colitis, serum SOD, GPx, and GSH decreased in all colitis groups. Honey, SP, combination regimen, and SSZ-treated groups experienced significant improvement in the SOD level when compared with the AA-colitis group. Administration of SP, the combination of honey and SP, mesalazine, and SSZ significantly increased GSH and GPx compared to the AA-colitis group. The serum TAC level of the AA-colitis group significantly decreased by 2.8-fold in comparison with the NC group.

### Histopathological Studies

**Figure 2:**
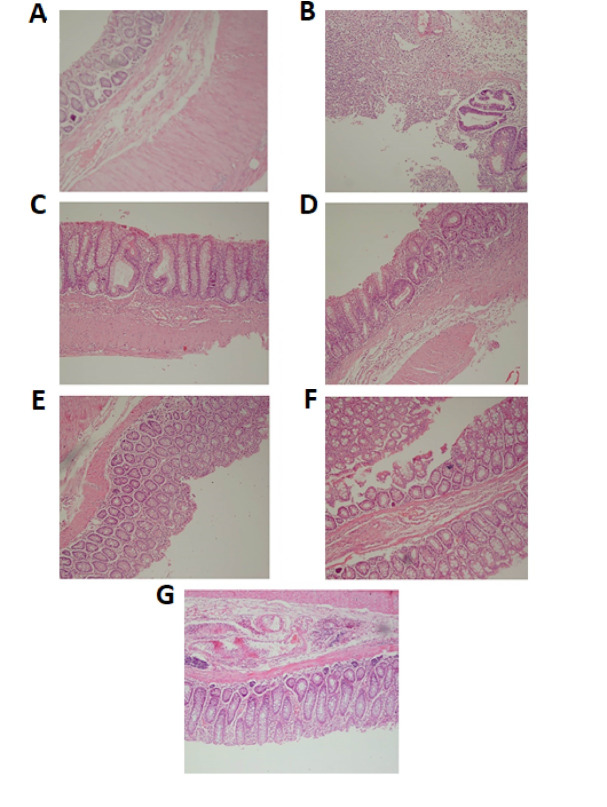
The histologic section of colon view (H&E, ×100)**. A:** Normal control group.** B:** Acetic acid-induced colitis group. **C:** Mesalazine group. **D:** Sulfasalazine group. **E:** Honey group.** F:** Spirulina platensis. G: S. platensis + Honey group

The histologic assessment of colitis based on severity, the extent of inflammation, the presence of ulceration, regeneration, crypt change, and depth of inflammatory cell infiltration revealed that in the AA-colitis-untreated group, ulceration, severity, and extent of inflammation were significantly higher than the treated colitis groups (P=0.04). Also, no sign of regeneration was observed in the untreated group. However, in treatment groups, regeneration and healing were comparable, but with no significant difference. As shown in Figure-2, the NC group showed normal colonic mucosa. The AA-colitis-untreated group showed mucosal ulceration along with crypt regeneration and goblet cell depletion. Mesalazine- and SSZ-treated groups showed near-complete mucosal regeneration with mild glandular disarray and architectural change, respectively. Restoration of normal colonic mucosa in honey SP, and SP + honey-treated groups was observed.

## Discussion

To the best of our knowledge, this study is the first to examine the therapeutic properties of oral honey, SP, and combination regimen in comparison with SSZ and mesalazine, as standard drugs, on experimental colitis. We showed that administration of honey and SP individually, and in combination, for seven consecutive days resulted in improvement in the TCS, colonic weight/length ratio, histological, and biochemical parameters in all treated colitis animals in comparison with the AA-colitis-untreated group. Ameliorative effects of honey and SP were comparable with SSZ and mesalazine. Our results confirm their potent antioxidant and anti-inflammatory properties and their efficacy to attenuate colonic tissue injury and oxidative damage.Our study has some novelty aspects in comparison with previous studies. First, this is the first study that has reported the therapeutic effects of honey on PGE2 and TAC when administered orally. Also, we are the first to assess the therapeutic effects of honey on serum IL-6, IL-1β, GSH, GPx, and SOD when administered orally. However, previous studies had reported colonic tissue levels of these parameters [17, 27, 28]. In addition, this study is the first to assess the therapeutic effects of oral administration of SP on tissue MPO, NO, and PGE2 levels, as well as serum GPx, SOD, IL-6, TNF-α, and IL-1β levels. Carotenoids are potent antioxidants, and their presence in spirulina is 10 times more than that in carrots [32]. Also, synergetic effects of tocopherols, ascorbic acid, phenolic acids, flavonoids, reduced glutathione, superoxide dismutase, catalase, Maillard reaction products, and other bioactive compounds result in antibacterial, anti-inflammatory, antioxidant, immunomodulatory, and analgesic activity of honey [33]. Intestinal edema led to increased colonic wet weight, which is a reliable index for severity and extent of colonic inflammation [30]. Epithelial cell necrosis, edema, and consequent increase in colonic weight resulted from increased generation of PGE2, IL-1β, TNF-α, and IL-6 [34]. Cyclooxygenase-2 (COX2) produces PGE2 and thromboxane B2, which stimulate edema and intestinal hyperemia [35]. Diminished production and release of NO, prostaglandin E2, TNF-α, and IL-6 can prevent edema and pain [33, 36]. In line with our study, Adeleye *et al*. reported the reduction of colonic weight after treatment with honey [29]. Honey and its flavonoids can significantly suppress leukocyte infiltration and ROS production. Their anti-inflammatory property is due to the inhibition of inducible nitric oxide synthase (iNOS) and COX2 expression, which leads to the reduced generation of IL-6, TNF-α, IL-1β, PGE2, and NO [33, 37]. Phenolic compounds can inhibit NO and PGE2, which results in suppression of edema [38]. In another study, oral selenium containing phycocyanin reduced the shortening of colon length [39]. Phycocyanin selectively inhibits COX2. It also reduces edema by hindering the production of PGE2 and TNF-α [25]. We did not find any study that had reported the therapeutic effects of SP and honey on TCS in experimental colitis. However, selenium-containing phycocyanin [39] and oral honey significantly decreased the disease activity index (DAI), which was in line with our TCS [17]. It has been shown that flavonoids in honey such as chrysin, naringenin, rutin, and quercitrin reduce the DAI in intestinal inflammation [40]. Flavonoids are known to have antidiarrheal effects. These antidiarrheal effects are attributed to their ability to improve tight junction barrier function, colonic absorptive function, and epithelial barrier permeability; suppress muscle contractility; reduce intestinal motility, and decrease intraluminal liquid [40]. In this study, the histological assessment revealed that in all treated colitis rats, regeneration and tissue repair occurred in the colon, which was comparable to standard drugs. Recent studies have shown that natural honey has a regenerative effect in degenerated tissue such as colonic epithelial cells by mobilizing endogenous stem cells, the proliferation of internal stem cell, and increasing blood flow to damaged tissues [17]. Honey releases hydrogen peroxide in a low concentration. It acts as an antimicrobial agent and contributes to wound healing and repairs damage through stimulating angiogenesis, cell proliferation, and the growth of epithelial and fibroblasts cells [41]. Besides, polyphenol compounds and bioactive molecules present in honey and spirulina such as carotenoids and C-phycocyanin synergistically help wound healing by stimulating cells proliferation and tissue regeneration; they also prevent cell death by scavenging free radicals [42, 43]. Our study showed that SP and the combination of SP and honey significantly decreased serum IL-6, TNF-α, and IL-1β, with similar efficacy to standard drugs. Also, we showed that honey, SP, and combination regimen reduced colonic PGE2 better than mesalazine. However, we did not find any study that had evaluated the therapeutic effects of oral SP on PGE2 and serum IL-6, TNF-α, and IL-1β levels. Previous studies have shown that SP suppressed the secretion and expression of proinflammatory cytokines through inhibition of NF-κB, and increased histone H3 acetylation [44-46]. This inhibitory effect of SP is attributed to its potent anti-inflammatory properties and antioxidants. Previous investigations demonstrated that β-carotene could inhibit COX-2, iNOS, TNF-α, and IL-1β mRNAs expression by repressing NF-κB activation. It also represses the transcription of IL-1β and IL-6 in stimulated macrophage [25]. Moreover, ergothioneine present in SP is known as a potent peroxynitrite, hypochlorous acid, and hydroxyl radical scavenger [47]. It can scavenge nonfree radical oxidizing species [48], decrease lipid peroxidation, and suppress IL-8, TNF-α, and IL-6 expression in epithelial cells [47]. Some studies have suggested that ergothioneine can maintain the proper ratio of GSH to oxidized glutathione and in vivo thiol defenses [47].Reduction of the MPO level of colonic tissue in our study showed the ability of honey, SP, and concurrent administration of honey with SP to inhibit neutrophil infiltration in inflamed colonic tissue similar to mesalazine and SSZ. Histological studies confirmed attenuated colon injury and inflammation. These observations were in line with previous studies on honey [27, 28]. However, we did not find any report on the therapeutic effects of SP on MPO. Kaempferol can suppress the adhesion and migration of neutrophils to endothelial [49]. Flavonoids such as quercitrin have anti-inflammatory properties verified by reducing infiltration of macrophages in damaged colonic tissue in rats [40]. One of the previous studies has shown that chrysin can control MPO activity and inflammatory responses in the mice colon [50]. The anti-inflammatory property of gallic acid, a phenolic compound in honey, is attributed to its ability to suppress iNOS, COX2; decline histamine release; and inhibit proinflammatory cytokines production [38]. Our results demonstrated that honey and SP effectively improve NO level in colitis. We did not find any study that had assessed the therapeutic effect of honey or SP on the colonic NO level. Nooh *et al*. reported the reduction in tissue iNOS level by administrating honey in colitis rats [17]. High levels of NO might react with the hydrosulfide group (−SH) of catalase, GSH, GPx, and SOD, which in turn can inactivate them [51]. These cellular antioxidants secure cells and tissues against harmful ROS and other free radicals [28]. Also, NO overproduction is associated with increased peroxynitrite formation that causes lipid peroxidation within the membranes and impairs colon integrity. MDA is a toxic end product of lipid peroxidation that causes cell death and tissue damage [35, 52]. Our study showed that honey, SP, and co-administration of SP and honey ameliorate MDA in colonic tissues similar to standard drugs. Similar results were reported after the administration of SP and honey individually in colitis rats [26-28]. It was reported that kaempferol reduced MDA concentrations in plasma [49]. Gallic acid reduces lipid peroxidation by scavenging free radicals [53]. The results of our study indicate the beneficial role of honey and SP in improving antioxidant status, confirmed by the increased GSH, GPx, SOD, and TAC levels in the treated groups compared to the AA-colitis untreated group. We did not find any study that had evaluated the therapeutic effect of honey on serum GSH, GPx, SOD, and TAC. Some previous studies measured GSH, SOD [17, 27, 28], and GPx in colonic tissue [27, 28]. Another study did not find a significant difference in colonic total antioxidant status (TAS) and GSH between the untreated colitis group in comparison with colitis rats treated with 2 g/kg spirulina [26]. Phycocyanin suppresses nicotinamide adenine dinucleotide phosphate oxidase activity resulting in the decline of ROS production and oxidative stress [54]. Its antioxidant property is 20-fold more than vitamin C [23]. Pinobanksin, a flavonoid within honey, plays a protective role against oxidative damage and can reduce MDA level, increase SOD activity, and glutathione levels [43]. A study reported that manuka honey could remove hydrogen peroxide and lipid hydroperoxides, maintain GSH in a reduced form, and protect GSH and GPx activity [15]. Our study indicated that the administration of honey and SP individually and in combination showed effectiveness. However, they showed no significant synergistic effects. Strengths of our study were the administration of two safe foods with antioxidant, wound-healing, and anti-inflammatory properties, and we examined more biochemical parameters than similar studies at the same time as histological changes. Due to budget constraints, our study had one limitation. We did not co-administer honey with the drugs of mesalazine and SSZ, as well as spirulina with mesalazine and SSZ simultaneously. More studies are required to investigate the effects of honey and spirulina in reducing the side effects of mesalazine and SSZ and increasing their efficacy in the future.

## Conclusion

Our results indicate that administration of honey and SP as natural and safe foods has a therapeutic effect on the experimental model of UC. Evidently, both of them reduced oxidative stress, secretion of proinflammatory cytokines, ameliorated histological colonic damage, and repaired the endogenous antioxidant defense system via their antioxidant and anti-inflammatory properties. These findings suggest that honey and SP might be effective and beneficial food choices in medical nutrition therapy in UC. However, further studies are recommended in human colitis.

## Acknowledgment

We are appreciative to the staff of the pathology laboratory of Shahid Faghihi Hospital for their assistance in performing the histological evaluation. We thank Mr. H. Argasi for his invaluable assistance in editing this manuscript. Shiraz University of Medical Sciences (grant no:94-7593) financed our investigation.

## Conflict of interest

None declared.

**Table 1:** Effects of Honey, Spirulina Platensis, and Spirulina + Honey on Biochemical Parameters of Colitis Rats. Values Expressed are Mean ± SEM.

**Biochemical parameters**	**Normal control**	**Acetic acid- induced colitis**	**Mesalazine**	**SSZ**	**Honey**	**SP**	**SP + Honey**
MPO (ng/mL)	7.55 ± 1.45*	20.10 ± .73	12.19 ± 1.22*	12.21 ± 1.26*	12.33 ± 1.19*	12.05 ±1.10*	12.59 ± 0.83*
NO(µM)	27.22 ± 2.79*	110.95 ± 15.63	42.03 ± 2.83*	44.02 ± 5.81*	41.76 ± 3.48*	53.54 ± 6.37	43.17 ± 4.02*
PGE2 (ng/mL)	0.39 ± 0.06*	2.94 ± 0.29	0.93 ± 0.18	0.58 ± 0.09*	0.55 ±0.06*	0.51 ± 0.07*	1.09 ± 0.36*
MDA (µM)	7.54 ± 1.55*	31.18 ± 3.56	6.34 ± 0.98*	6.87 ± 0.77*	7.36 ± 1.43*	5.62 ± .91*	6.29 ± 1.08*
IL -1β pg/mL serum	24.75 ± 2.29*	105.20 ± 11.69	39.61 ± 2.70*	42.80 ± 5.26*	43.55 ± 2.75**	39.53 ± 3.42*	40.08 ± 3.72*
IL-6 (pg/mL)	40.23 ± 4.45*	202.02 ± 16.54	76.06 ± 10.50*	74.84 ± 6.8^5^*	82.97 ±10.45	68.09 ± 7.5^3^*	70.79 ±8.02*
TNF-α (pg/mL)	23.93 ± 3.25*	45.58 ± 2.19	28.51 ± 3.09*	28.68 ± 2.18*	37.21 ± 4.33	28.23 ± 3.22*	28.57 ± 3.02*
SOD(U/mL)	64.61 ± 3.62*	31.82 ± 2.84	51.12 ± 5.5	58.43 ± 2.7*	57.09 ± 3.47*	58.13 ± 2.82*	59.15 ± 2.33*
GPx(U/mL) serum	87.84 ± 4.48*	57.03± 1.48	90.00 ± 11.08*	86.71 ± 5.09*	76.87 ± 2.35	87.58 ± 8.78*	95.10 ± 6.49*
GSH(mmol/L)	0.22 ±0.03*	0.08 ± .004	0.14 ± 0.01*	0.13 ± 0.006*	0.12 ± 0.01	0.15 ±0.02*	0.14 ± 0.01*
TAC(mM)	0.31 ±0.05*	0.11 ± .004	0.23 ± 0.02*	0.22 ± 0.02*	0.16 ± 0.003	0.21 ± 0.01*	0.23 ± 0.02*

**MPO:** Myeloperoxidase; **NO:** Nitric oxide; **PGE2:** Prostaglandin E2; **MDA:** Malondialdehyde;** IL:** Interleukin; **TNF-α:** Tumor necrosis factor-α; **SOD:** Superoxide dismutase, **GPx:** Glutathione peroxidase; **GSH:** Glutathione; **TAC:** Total antioxidant capacity; **SSZ:** Sulfasalazine; **SP:** Spirulina platensis * P<0.05 vs. acetic acid-induced colitis group, **P<0.05 vs. normal control group
